# 509. Role of cell-mediated immune monitoring using Interferon-γ Enzyme-linked Immunosorbent Spot Assay to predict CMV infection within six months after kidney transplantation

**DOI:** 10.1093/ofid/ofac492.565

**Published:** 2022-12-15

**Authors:** Warunyu Namsiripongpan, Surasak Kantachuvesiri, Jackrapong Bruminhent

**Affiliations:** Faculty of Medicine Ramathibody Hospital, Mahidol University, Bangkok, Krung Thep, Thailand; Faculty of Medicine Ramathibody Hospital, Mahidol University, Bangkok, Krung Thep, Thailand; Faculty of Medicine Ramathibodi Hospital, Mahidol University, Bangkok, Krung Thep, Thailand

## Abstract

**Background:**

CMV infection can cause substantial morbidity and mortality in kidney transplant (KT) recipients due to an impairment in cell-mediated immunity (CMI) from immunosuppressive drugs. We investigated the role of CMI monitoring prior to and after transplant to predict CMV infection after KT.

**Figure:**
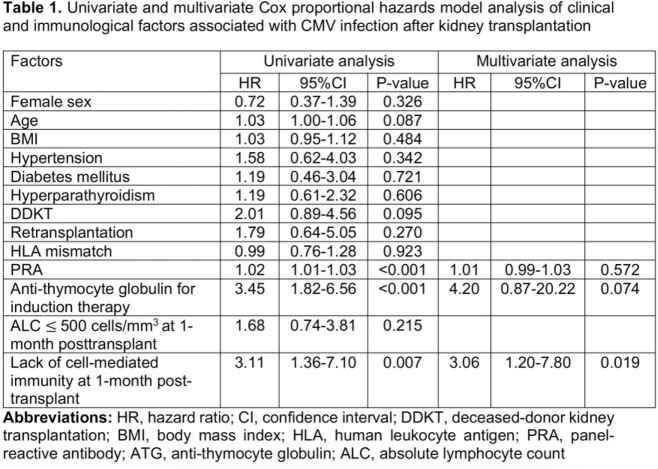

**Methods:**

A prospective study was performed between December 2020 and December 2021. All adult KT recipients underwent CMI measurement by investigating IFN-γ-producing T cells using enzyme-linked immunosorbent (ELISpot) assay before and one month post-transplant. The incidence of CMV infection within six months after transplant was reported, and predictors of CMV infection were analyzed using the Cox proportional hazard model.

**Results:**

We included 93 KT recipients with a mean (SD) age of 44 (11) years; 59.1% were male, and 98.9% were CMV seropositivity. Twenty-two (23.7%) participants received anti-thymocyte globulin (ATG) for induction therapy. A median (IQR) of IFN-γ-producing T cells measured one month after transplant was significantly lower compared to before transplant (148 [54-389] vs. 763 [409-1,067] SFUs per 2.5 x 10^5^ PBMCs, p < 0.001). Forty (42.9%) KT recipients who developed CMV infection appeared to have significantly less IFN-γ-producing T cells compared to those did not develop CMV infection (47.1%) (115 [33-237] vs. 238[76-492] SFUs/2.5x10^5^ PBMCs, p=0.019). In univariate analysis, predictors for CMV infection included higher panel-reactive antibody (HR 1.02 [95%CI, 1.01-1.03], p< 0.001), receiving ATG for induction therapy (HR 3.45 [95%CI, 1.82-6.56], < 0.001) and lack of CMI one month after transplant defined as IFN-γ-producing T-cells of < 250 SFUs/2.5x10^5^ PBMCs (HR 3.11 [95%CI, 1.36-7.10], p=0.007). In multivariate analysis, lack of CMI one month after transplant is the only predictor which remained independently associated with CMV infection (HR 3.1 [95%CI 1.2-7.80], p=0.019) (Table 1).

**Conclusion:**

KT recipients with low IFN-γ-producing T cell responses are more likely to develop CMV infection post-transplant. Quantification of CMI using ELISpot assay could potentially predict those at risk of CMV infection after KT.

**Disclosures:**

**All Authors**: No reported disclosures.

